# Global Experience With Rotavirus Vaccines

**DOI:** 10.1093/infdis/jiab399

**Published:** 2021-08-10

**Authors:** Rachel M Burke, Jacqueline E Tate, Umesh D Parashar

**Affiliations:** Viral Gastroenteritis Branch, US Centers for Disease Control and Prevention, Atlanta, Georgia, USA

**Keywords:** rotavirus, rotavirus vaccines, vaccine effectiveness, pediatric gastroenteritis, vaccine-preventable disease, acute gastroenteritis

## Abstract

Rotavirus is a major cause of severe pediatric diarrhea worldwide. In 2006, 2 live, oral rotavirus vaccines, Rotarix and RotaTeq, were licensed for use in infants and were rapidly adopted in many high- and middle-income settings where efficacy had been demonstrated in clinical trials. Following completion of successful trials in low-income settings, the World Health Organization (WHO) recommended rotavirus vaccination for all infants globally in 2009. In 2018, 2 new rotavirus vaccines, Rotasiil and Rotavac, were prequalified by WHO, expanding global availability. As of March 2021, rotavirus vaccines have been introduced nationally in 106 countries. Since, Rotavirus vaccines have demonstrated effectiveness against severe disease and mortality, even among age groups in eligible for vaccination. Cross-genotypic protection has been demonstrated, and the favorable benefit-risk profile of these vaccines continues to be confirmed. Ongoing research seeks to better understand reasons for the geographic disparities in effectiveness observed, in order to optimize vaccine strategies worldwide.

Rotavirus is a leading cause of severe pediatric diarrhea worldwide, estimated to have caused 258 million diarrheal episodes and >128 000 associated deaths among children <5 years of age in 2016 [1]. The mortality burden of rotavirus falls most heavily on developing countries where access to healthcare is suboptimal [1, 2]. Rotavirus has been estimated to be the leading cause of pediatric diarrheal deaths in countries with low- to high-middle sociodemographic index (SDI), and as the third leading cause of pediatric diarrheal deaths in high SDI countries [3]. The proportion of rotavirus illness among infants and young children hospitalized for severe diarrhea prior to widespread introduction of rotavirus vaccine has been found to be similar across geographies and in some studies was highest in the highest industrialization strata [4, 5], suggesting that traditional measures to improve hygiene and sanitation and access to safe water are unlikely to fully control the disease. Initial rotavirus infections occur early in life, and in the prevaccine era, nearly all children suffered at least 1 rotavirus infection by the age of 5 years [4]. Rotavirus infection confers partial immunity, with the level of protection against disease increasing with each subsequent infection [6]. Vaccination, through mimicking the effects of natural rotavirus infection, is considered the best means of control of rotavirus disease [7, 8].

## EVIDENCE AND IMPACT OF THE FIRST 2 GLOBALLY LICENSED ROTAVIRUS VACCINES: ROTARIX AND ROTATEQ

In 2006, 2 live, oral rotavirus vaccines, Rotarix (GlaxoSmithKline) and RotaTeq (Merck), were licensed for use in infants based on data from trials conducted in the United States, Europe, and Latin America [9, 10]. Due to the experience with RotaShield, a tetravalent reassortant rhesus rotavirus vaccine that was withdrawn from the US market in 1999 because it carried a risk of 1 additional case of intussusception (a form of bowel obstruction) per 10 000 vaccinated infants, large clinical trials for both Rotarix and RotaTeq (60 000–70 000 infants each) were conducted to examine safety [11]. Neither vaccine was found to cause an increased risk of intussusception in clinical trials, and both vaccines were highly efficacious against severe rotavirus gastroenteritis [9, 10]. On the strength of these data, in 2006 the World Health Organization (WHO) recommended rotavirus vaccines for use in high- and middle-income settings [12]. However, because of concerns about the efficacy of oral rotavirus vaccines in low-income settings, WHO recommended additional trials to examine vaccine efficacy in these settings [12].

Rotavirus vaccine uptake in the Americas was rapid, and evidence quickly accumulated on the effectiveness of these vaccines in reducing hospitalizations and deaths from pediatric gastroenteritis [13–19]. In the United States, an analysis of hospital discharge data showed significant reductions in rotavirus hospitalizations in 2008 as compared to prevaccine years, even among older children and young adults 3–24 years of age, who would not have been vaccinated [14]; similar results were seen when the analysis was extended to 2010 [15]. These findings, which suggested that rotavirus vaccination may also offer indirect protection to older children and adults by reducing overall levels of community transmission of rotavirus, provided important evidence of the far-reaching benefits of rotavirus vaccine introduction. The potential life-saving impact of rotavirus vaccination was first demonstrated in Mexico, where an analysis of pediatric diarrheal mortality found significant declines after the introduction of rotavirus vaccine [16, 19].

One question that would urgently impact the viability of rotavirus vaccines as a means of disease control was the extent to which these vaccines could provide cross-genotype protection, particularly against strains not included in the vaccine. Rotavirus genotypes are defined by 2 outer capsid proteins: VP4 (which defines the G type) and VP7 (which defines the P type) [20, 21]. Although a limited number of strains tend to account for the bulk of infections (G1P[8], G2P[4], G3P[8], G4P[8], G9P[8], and G12P[8]), there are numerous other strains that circulate at a lower frequency, and overall circulating patterns vary geographically and over time [22, 23]. Rotarix is based on an attenuated G1P[8] human rotavirus, whereas RotaTeq is a pentavalent vaccine containing bovine-human reassortant virus with the G1, G2, G3, G4, and P[8] antigens [9, 10]. Encouragingly, data from a US-based active surveillance platform demonstrated high vaccine effectiveness of both vaccines against a variety of strains, including nonvaccine-type strains [17, 24]. Similarly, data from an efficacy trial in Africa found Rotarix efficacy to be comparable across vaccine- and nonvaccine-type strains [25]. Results from postlicensure evaluations in Latin America further confirmed the cross-protection conferred by vaccination [26–29].

**Figure 1.**
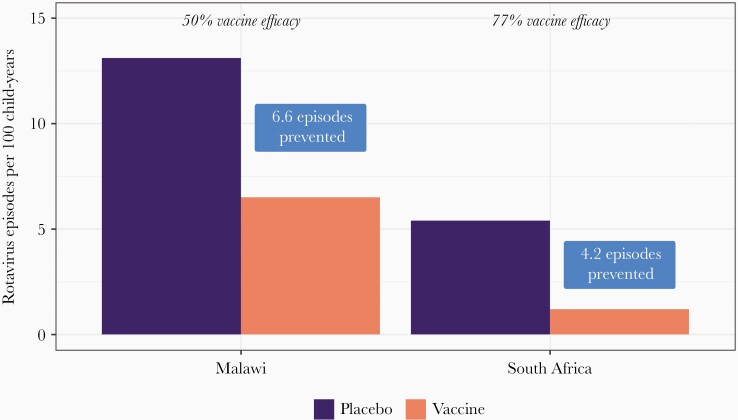
Vaccine impact (episodes prevented) and vaccine efficacy by country: Malawi and South Africa. Adapted from Madhi et al 2010 [25] analysis of clinical trial data collected 2005–2007.

In 2009, after the completion of additional clinical trials showing vaccine efficacy in low-income countries of Africa and Asia, WHO recommended rotavirus vaccines for inclusion in national immunization programs worldwide [7]. This expansion opened up rotavirus vaccine recommendations to areas most in need of intervention, as it has been estimated that 65% of rotavirus deaths occur in just 10 countries—all in Africa and Asia [2]. Vaccine efficacy in clinical trials of Rotarix and RotaTeq in Asia and Africa ranged from 51% to 64%, moderate in comparison to results from the initial trials in high-income countries, in which efficacy was >85% [9, 10, 25, 30, 31]. However, given the higher burden of rotavirus disease in these settings, even a vaccine with modest efficacy can have a substantial public health impact. As an example, when comparing results from Malawi, a lower-resource country, with those from South Africa, a higher-resource country, it is clear that despite lower efficacy in Malawi, the health impact in terms of episodes of severe rotavirus disease averted by vaccination of 100 infants was greater in Malawi as compared to South Africa, which had a higher estimated efficacy [25] (Figure 1). The effectiveness and public health impact of these vaccines continues to be borne out in postintroduction evaluations, with evidence suggesting that although effectiveness tends to be lower in countries with high all-cause child mortality burden, impact of rotavirus vaccine in terms of reducing rotavirus-associated hospitalizations and deaths tends to be greater in these high child-mortality countries as compared to other settings [32, 33].

**Figure 2.**
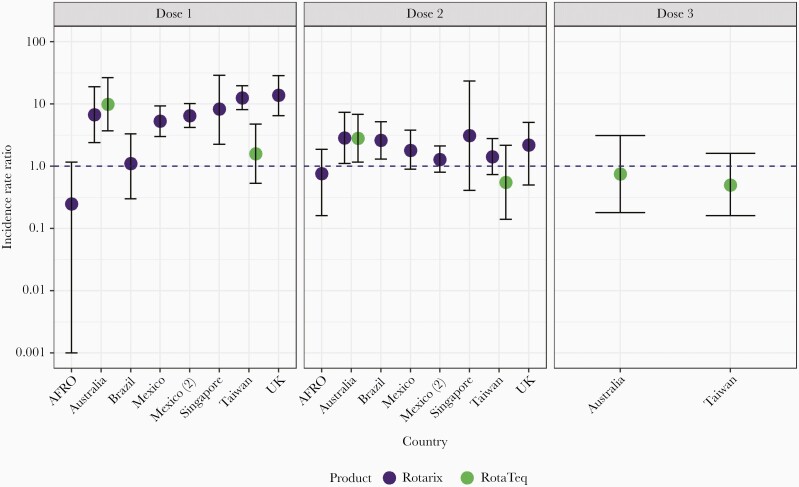
Estimates of incidence rate ratio for intussusception in the 1–7 days following rotavirus vaccine administration, using the self-controlled case series method, by country and dose [34, 36, 39, 40–42]. Study data from 2006 through 2016. Dots indicate point estimates for incidence rate ratios, and lines and whiskers indicate 95% confidence intervals. Abbreviations: AFRO = African Rotavirus Surveillance Network Countries (Ethiopia, Ghana, Kenya, Malawi, Tanzania, Zambia, and Zimbabwe); UK = United Kingdom.

To further support expansion of Rotarix and RotaTeq into additional national vaccination programs, more evidence was needed on the safety of these 2 vaccines during routine use. Although no evidence of an association with intussusception was noted during clinical trials, these studies were not powered to detect an increased risk smaller than 1 per 10 000 children vaccinated. Postintroduction evaluations in several high- and middle-income countries found an even smaller but significant risk of intussusception associated with both vaccines, approximately 1–6 additional cases per 100 000 infants vaccinated [34–42]. However, as the benefits of vaccination still exceeded the possible intussusception risk [43], no changes were made to overall recommendations. Furthermore, later analysis of data from 7 African countries using Rotarix found no evidence of a significantly increased risk of intussusception after vaccination [44], nor did another analysis of data from South Africa [45] (Figure 2). These analyses reaffirm the favorable safety profile of rotavirus vaccines and suggest that the benefits of rotavirus vaccination far outweigh the associated risks.

## EXPANDING GLOBAL ROTAVIRUS VACCINE CHOICE: IMPLEMENTATION OF ROTAVAC AND ROTASIIL

In 2018, interruptions to the global rotavirus vaccine supply underscored the importance of having multiple affordable vaccine options available to countries. That same year, 2 Indian-manufactured rotavirus vaccines were prequalified by WHO: Rotasiil (Serum Institute) and Rotavac (Bharat Biotech), both live, oral vaccines given in a 3-dose infant schedule. Rotasiil is based on a bovine-human reassortant strain and contains G1, G2, G3, G4, and G9 antigens, while Rotavac is based on a naturally occurring neonatal strain of G9P[11] [46, 47]. These vaccines have several features that make them attractive to many countries: Rotasiil is heat-stable for extended periods of time at high ambient temperatures, while Rotavac requires only a 5-drop dose, and both vaccines are available at a relatively low cost compared to Rotarix and RotaTeq [48, 49]. Clinical trials for these vaccines were conducted in India and Niger, and both demonstrated similar efficacy as was seen for Rotarix and RotaTeq in Asia and Africa [47, 50–52]. Both vaccines are in routine use in India, and each has also been adopted in a handful of other countries in Africa and Asia [53]. One country where a substantial impact might be expected is the Democratic Republic of Congo, which has both a large birth cohort and a high burden of rotavirus disease [2, 54, 55]. Given that Rotavac and Rotasiil were prequalified only in 2018, the evidence base for safety and effectiveness in routine usage is still being built. However, several postlicensure evaluations of Rotavac usage in India have been published, all showing no increased risk of intussusception associated with vaccination [56–58]. Additional evaluations of Rotavac and Rotasiil are underway to further assess the safety and effectiveness of both vaccines under real-world conditions of use.

## NATIONAL VACCINES: ROTAVIN AND LANZHOU LAMB ROTAVIRUS VACCINE

In addition to Rotasiil and Rotavac, 2 other indigenously produced rotavirus vaccines are licensed and in use in their countries of origin: Rotavin (POLYVAC) is licensed for use in Vietnam, and Lanzhou Lamb Rotavirus Vaccine (LLRV; Lanzhou Institute of Biological Products) is licensed for use in China [59–63]. Although neither vaccine is currently part of any national immunization program, both are available on the private market in their respective countries of origin. Rotavin has demonstrated immunogenicity in a phase 2 clinical trial and has been introduced into the immunization schedule in selected areas of 2 provinces, where its impact and effectiveness are being evaluated [59]. A phase 3 trial of a liquid presentation of the vaccine has been completed, and results are pending publication [64]. No efficacy data are available for LLRV, but vaccine effectiveness estimates have varied from 35% to 77% [63, 65–68]. Nationally produced vaccines are an important element in ensuring broad global access to affordable vaccines, and more data on these 2 nationally licensed products will inform the global research agenda.

## RESEARCH GAPS AND FUTURE DIRECTIONS

Recently, there has been increased scientific interest in possible off-target effects of rotavirus vaccination. The most well-documented such effect is a decrease in seizure hospitalizations following rotavirus immunization, which has been observed in several countries through both cohort studies and ecological analyses [69–73]. It is hypothesized that this effect is mediated through reduction in rotavirus disease, which has been shown to cause seizures in addition to (or sometimes in the absence of) gastrointestinal illness [74, 75]. Two autoimmune diseases, celiac disease (CD) and type 1 diabetes (T1D) have also been linked to rotavirus infection, although evidence suggests that the etiology of both autoimmune conditions is multifactorial [74, 76]. Two studies have found that rotavirus vaccination may have some protective effect against CD, in conjunction with other factors [77, 78]. Although 2 analyses have found rotavirus vaccination to be associated with reduced T1D diagnosis [79, 80], several other studies have shown no significant effect [77, 81–83]. Further research will be necessary to better elucidate possible relationships of rotavirus vaccination to CD and T1D, as well as to identify any other possible unanticipated benefits of rotavirus vaccination [74–76].

Another area of ongoing research involves the differential effectiveness of rotavirus vaccine by setting, whereby higher effectiveness is demonstrated in higher-income settings as compared to lower-income settings. Although this phenomenon has been well documented, the exact reasons for this disparity, and the best interventions, are still not well defined. Multiple possible factors have been identified, such as those that may act directly on vaccine virus in the gut (eg, maternal antibodies, breast milk, stomach acid, and oral polio vaccine [OPV]), as well as factors that act to impair general immune response (eg, malnutrition, microbiome, and coinfections such as human immunodeficiency virus [HIV]) [84–92]. Available evidence suggests that delaying rotavirus vaccination schedules may contribute to enhanced immune response due to waning interference from maternal antibodies [93], but it is not known if this would translate into increased effectiveness, and any changes to vaccine schedules must also be balanced with practical and logistical concerns, as well as the desire to protect infants early in life. Similarly, while OPV has been well documented to interfere with rotavirus vaccine immunogenicity when coadministered (the converse does not occur) [85], this finding does not suggest a clear public health intervention. Several interventions specific to the time of vaccination have been tested, for instance withholding breastfeeding and adding nutritional supplementation, but these were found to have little or no effect on rotavirus vaccine immune response [88, 94]. Research also suggests that susceptibility to rotavirus (and thus live rotavirus vaccines) varies by histo-blood group antigens in a rotavirus P-genotype–dependent way; the expression of these antigens is governed by polymorphisms in 2 genes, the prevalence of which varies by population [95–107]. As a specific example, the genotype conferring increased susceptibility to P[6] rotaviruses is more common in Africa, a setting where both increased circulation of P[6] rotaviruses and moderate efficacy of current rotavirus vaccines have been observed. Taken together, the evidence suggests a need for holistic interventions, such as those that could improve overall infant nutritional status, and a potential role for parenterally administered rotavirus vaccines, which would not be subject to some of the same limitations as the current oral vaccines.

Indeed, there are several rotavirus vaccines under development that are designed for parenteral administration; another candidate that has completed phase 2 trials is being developed for neonatal administration [108]. The most advanced parenteral vaccine candidates are 2 subunit vaccines: one contains the P[8] antigen, and the other contains P[4], P[6], and P[8] antigens, both being developed by PATH and both demonstrating immunogenicity [109, 110]. RV3-BB, the rotavirus vaccine candidate being developed for neonatal administration, is based on a naturally attenuated neonatal strain of G3P[6] rotavirus that was initially isolated in Australia [111, 112]. A phase 2b trial demonstrated both safety and efficacy of this vaccine when given on a schedule that includes a birth dose (along with doses at 8 and 14 weeks of age) as well as a more standard infant schedule at 8, 14, and 18 weeks of age (although the trial was not powered to detect extremely rare side effects such as intussusception) [111].

## CONCLUSIONS

**Figure 3.**
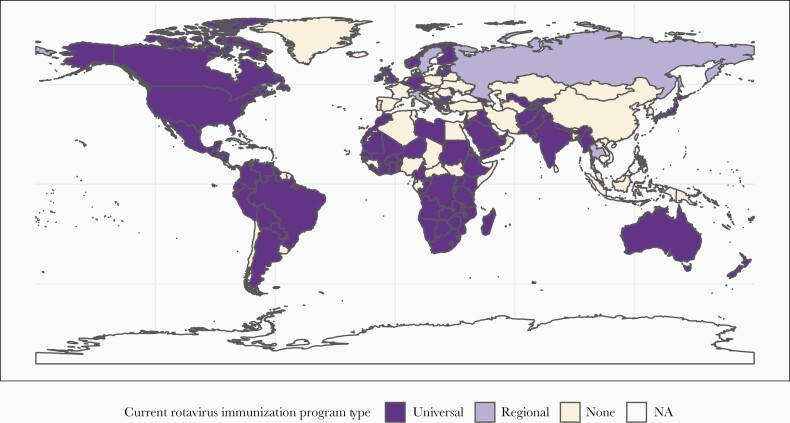
Map of rotavirus vaccine introductions by country, with program status (universal vs regional vs none).

As of March 2021, rotavirus vaccines have been introduced nationally in 106 countries, and regionally within 4 countries (Figure 3) [53]. It is not yet clear what impact the coronavirus disease 2019 (COVID-19) pandemic may have had on rotavirus vaccine coverage or burden, but encouragingly, despite the pandemic, several countries introduced rotavirus vaccines into their national schedules during 2020. Although great progress has been made over the past 15 years, there is a notable gap evident: in much of Asia, an area with a substantial rotavirus burden, no national immunization program is in place. A 2018 analysis estimated that early introduction of rotavirus vaccines throughout Asia could have averted >700 000 hospitalizations and approximately 35 000 deaths among children <5 years of age, with impact concentrated in the highest-burden countries [113]. As we look forward towards the next 15 years of rotavirus vaccines, our goal must be to close these regional gaps to protect more children from severe gastroenteritis. With multiple planned introductions in 2021, including in Bangladesh, a country with an extraordinarily high rotavirus burden [114], we can look forward to continued progress in improving child health.

## Notes

*Potential conflicts of interest.* **All authors: No reported conflicts of interest. All authors have submitted the ICMJE Form for Disclosure of Potential Conflicts of Interest. Conflicts that the editors consider relevant to the content of the manuscript have been disclosed.**

Presented in part: 15th International Conference on Diarrheal Disease and Nutrition, 28–30 January 2020, Dhaka, Bangladesh.
